# A low BUN/creatinine ratio predicts histologically confirmed acute interstitial nephritis

**DOI:** 10.1186/s12882-023-03118-0

**Published:** 2023-03-27

**Authors:** López Giacoman Salvador, González Fuentes Carolina, Robles Dávila Jesús, Soto Abraham María Virgilia, Román Acosta Susana, Chávez Íñiguez Jonathan, Salas Pacheco José Luis, Ronco Claudio

**Affiliations:** 1 Division of Nephrology, Department of Medicine. Hospital General ISSSTE Zacatecas. Zacatecas, México. Adolfo Lopez Mateos Blvd Without Number, Zacatecas, Mexico; 2 Department of Medicine. Hospital General de Zacatecas, Division of Nephrology, Zacatecas, México; 3 Department of Nephropathology, Instituto Nacional de Cardiología Ignacio Chávez. , Mexico City, México; 4 Department of Nephrology. Hospital Civil de Guadalajara, Fray Antonio Alcalde, Jalisco, México; 5 Department of Cardiology, Centenario Hospital Miguel Hidalgo, Aguascalientes, México; 6 Department of Nephrology, Dialysis and Kidney Transplant, International Renal Research Institute, San Bortolo Hospital, Vicenza, Italy

**Keywords:** Acute interstitial nephritis, Acute kidney injury, BUN, Creatinine

## Abstract

**Introduction:**

In hospitalized patients with acute renal injury (AKI), acute tubulointerstitial nephritis (AIN) constitutes one of the leading etiologies. The objective of this study was to identify clinical and biochemical variables in patients with AKI associated with kidney biopsy-confirmed AIN.

**Methods:**

For our prospective study, we recruited hospitalized patients aged 18 years and older who were diagnosed with AKI based on biochemical criteria. Prior to enrollment, each patient was assessed with a complete metabolic panel and a kidney biopsy.

**Results:**

The study consisted of 42 patients (with a mean age of 45 years) and equal numbers of male and female patients. Diabetes and hypertension were the main comorbidities. Nineteen patients had histological findings consistent with AIN. There was a correlation between histology and the BUN/creatinine ratio (BCR) (r = -0.57, *p* = 0.001). The optimal Youden point for classifying AIN via a receiver operating characteristic (ROC) curve analysis was a BCR ≤ 12 (AUC = 0.73, *p* = 0.024). Additionally, in diagnosing AIN, BCR had a sensitivity of 76%, a specificity of 81%, a positive predictive value of 81%, a negative predictive value of 76%, and OR of 14 (95% CI = 2.6 to 75.7, *p* = 0.021). In the multivariable analysis, BCR was the sole variable associated with AIN.

**Conclusion:**

A BCR ≤ 12 identifies AIN in patients with AKI. This study is the first to prospectively assess the relationship between renal biopsy results and BCR.

## Introduction

Acute interstitial nephritis (AIN) accounts for 13–27% of acute kidney injury (AKI) cases in hospitalized patients and is diagnosed in 13% of kidney biopsies performed for AKI [1–3]. AIN histopathology is characterized by interstitial inflammation and tubulitis. Interstitial infiltrates predominantly contain lymphocytes and monocytes, but plasma cells, neutrophils, and histiocytes may also be present [1, 2]. AIN has become increasingly relevant in acute and critical care settings over the last few years [2]. Additionally, AIN could be a potential origin of chronic kidney disease from unidentified etiology [4].

The classic triad described for AIN presentation includes fever, dermatosis, and eosinophilia. Eosinophilia is only present in 10% of patients [5], thus resulting in poor diagnostic performance [6, 7]. Previous studies have evaluated novel biomarkers of AIN by analyzing markers of inflammation, interstitial edema, cellular damage, and tubular lesions; however, their clinical utility remains unknown [8]. Despite the many examined biomarkers, the gold standard continues to be percutaneous renal biopsy [9, 10]. It is widely accepted that the blood-ureic nitrogen to creatinine ratio (BCR) decreases in renal tubular lesions, but prospectively obtained clinical-histological evidence relating low BCR to AIN is lacking [6, 11]. Therefore, this study aimed to determine classic clinical and biochemical predictors (specifically BCR) associated with histopathologically-confirmed AIN in patients with AKI.

## Methods

From June 2018 to June 2019, we prospectively recruited hospitalized patients of both sexes aged 18 years and older and diagnosed them with AKI according to the creatinine criteria established by the Kidney Disease: Improving Global Outcomes guidelines [12]. The study exclusion criteria included pregnancy, acute coronary syndrome, advanced liver disease, rhabdomyolysis, previous glucocorticoid treatment, and sepsis. The comprehensive clinical evaluation recorded prescribed and over-the-counter medications, as well as the consumption of herbal medication. An exhaustive physical examination evaluated cutaneous rash and other signs of systemic diseases. Peripheral blood samples were obtained for a complete blood count and a basic metabolic panel. Moreover, a biochemical urine analysis and spot proteinuria analysis were also performed.

This study was approved by the Institutional Review Board and was conducted in adherence to the Helsinki Declaration. The protocol followed the Strengthening the Reporting of Observational Studies in Epidemiology (STROBE) guidelines. Informed consent was obtained from all of the patients.

For determining the variables, the nephrotic syndrome was defined as proteinuria > 3.5 g per day, serum albumin < 2.5 g/dL, and clinical evidence of peripheral edema and hyperlipidemia [13]. The nephritic syndrome was defined as oliguria, hematuria with red blood cell casts, subnephrotic proteinuria, and hypertension [14]. The degrees of interstitial fibrosis and arteriosclerosis were reported in accordance with the standardized grading proposed by Sethi et al [15].

### Percutaneous kidney biopsy

An experienced nephrologist performed all of the kidney biopsies. Prior to the procedure, all the patients were normotensive, and kidney size and anatomy were evaluated via ultrasound. Following the localization of the left kidney inferior pole, the puncture site was marked, the skin was prepared with chlorhexidine, and 1% lidocaine was used for local anesthesia. The biopsy procedure was guided by real-time ultrasound and was performed by using an automatic biopsy gun (Magnum, Bard Medical) with a disposable, 18-gauge core needle. Two kidney core biopsies were obtained from each patient. After the procedure, the patients were closely monitored for 24 h.

### Tissue processing and analysis

All of the tissues were uniformly processed in the same nephropathology laboratory and analyzed by a single nephropathologist. Of the two core biopsies that were obtained per patient, one specimen was fixed in 10% formaldehyde, embedded in paraffin, and stained with hematoxylin and eosin, PAS, Masson’s trichrome, and Jone’s methenamine silver. The second sample per patient was placed in Zeus media tissue fixative, after which it was washed, rehydrated, and frozen at –24 °C for cryosectioning and direct immunofluorescence staining. Sections were stained with antibodies against IgG, IgA, IgM, Ciq, C3c, C4c, fibrinogen, albumin, kappa, and lambda.

Histologically, AIN was defined as interstitial edema and interstitial infiltrate consisting primarily of mononuclear or polymorphonuclear leukocytes. Tubulitis was defined as the invasion of the tubular basement membrane by inflammatory cells [13].

### Statistical analysis

In accordance with their respective distributions, continuous variables were expressed by the mean ± standard deviation or the median with interquartile range. The normality of the distribution was evaluated by using the Kolmogorov‒Smirnov test. The chi-square test was performed to evaluate the association between the qualitative variables. Differences between groups were assessed with the Student’s t-test or the Mann‒Whitney U test (according to the distribution).

A multivariate analysis was performed with binary logistic regression. The variables with a *p*-value < 0.1 in the bivariate analysis were selected for inclusion as the explanatory variables within the multivariable model. The dependent variable was dichotomous and expressed as the presence or absence of histologically confirmed AIN. The independent variables that were included in the model were BUN, serum creatinine, BCR, and blood glucose concentration.

The optimal BCR value for discriminating AIN was identified by using a ROC analysis. The Youden point was selected to maximize the sensitivity over the specificity. The bilateral p-value for statistical significance was established at < 0.05. The statistical analysis was performed by using R (version 4.04) in the R-Studio.

## Results

**Fig. 1:**
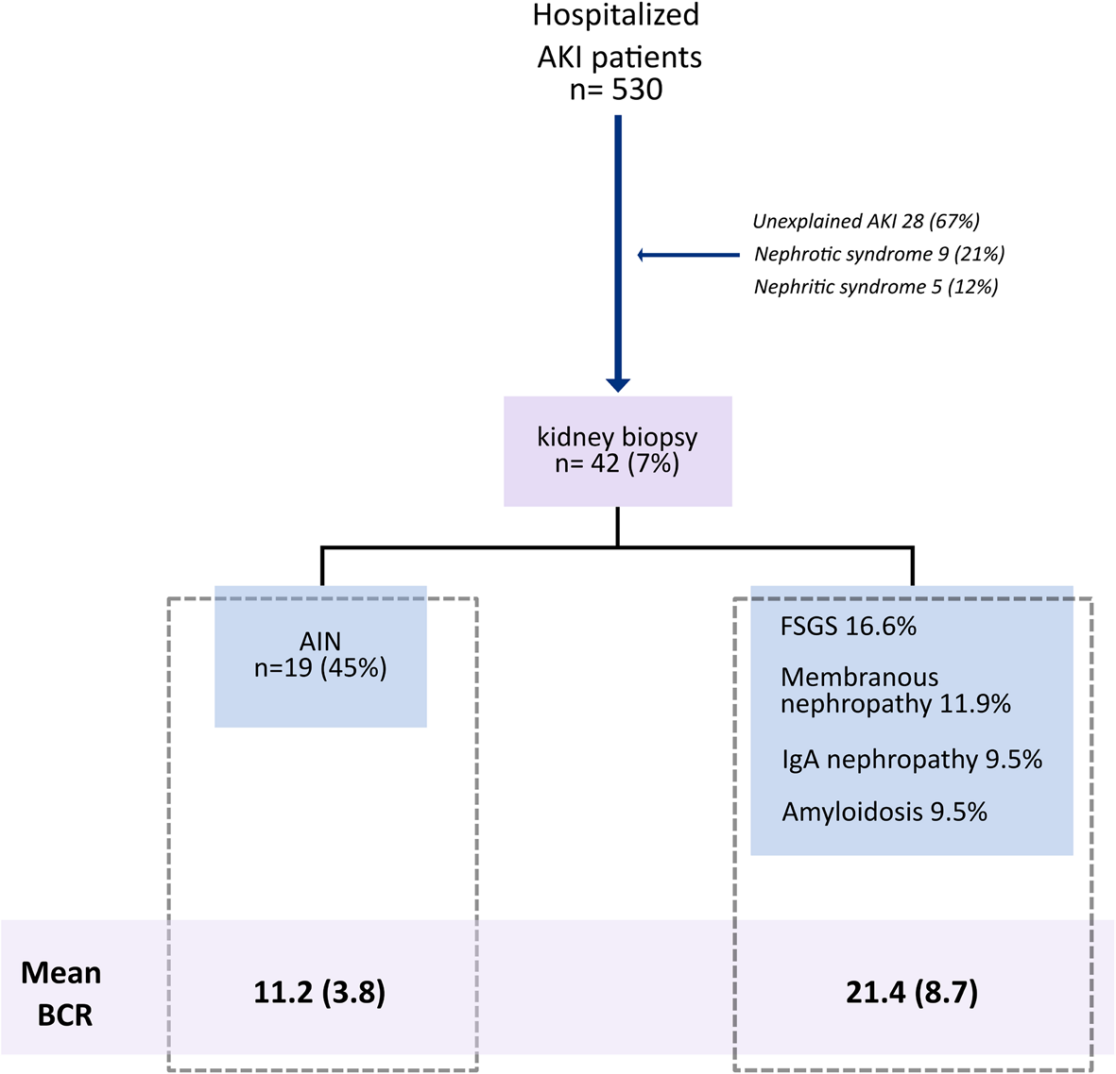
Flowchart showing the study patient distribution. Forty-two individuals required percutaneous kidney biopsy. In nearly half of AIN patients, the etiology was acute kidney injury. In patients with AIN, the BCR was significantly lower. AIN = acute interstitial nephritis, AKI = acute kidney injury, BCR = BUN/creatinine ratio, FSGS = focal and segmentary glomerulosclerosis

Five hundred thirty patients diagnosed with AKI were assessed, of whom 42 (7%) patients satisfied the inclusion criteria and underwent a percutaneous renal biopsy (Fig. 1). Table 1 describes the demographic and biochemical characteristics of the patient cohort. The mean age was 45 years, and half of the patients were female. The main comorbidities were hypertension and diabetes (33% and 17%, respectively). In addition, the biopsy indications were unexplained AKI (66%), nephrotic syndrome (21%), and nephritic syndrome (12%). There were no complications from the biopsy procedures. The glomerular disease was reported in twenty-three patients (54%), the primary reported glomerular disease was focal and segmental glomerulosclerosis (FSGS) in 16.6% of patients, followed by membranous nephropathy at 11.9%, Ig A nephropathy at 9.5%, and amyloidosis at 9.5%. Among the study participants, AIN was observed in 19 (45%) patients. Nodular arteriolopathy was reported in 28% of patients, followed by arteriosclerosis in 17% of the patients. Half of the samples had interstitial fibrosis, 47%, of which presented with third-degree fibrosis.

**Table 1:** Demographic characteristics

Variable	All *n* = 42	AIN	No AIN	*p*
		***n* = 19**	***n* = 23**	
**Gender**				
male	21	11	10	0.3
female	21	8	13	
Age (years)	44 (± 19)	45 (25–53)	40 (29–53)	0.3
Hypertension	14 (33%)	7 (37%)	7 (30%)	0.6
Diabetes	7 (16%)	4 (21%)	3 (13%)	0.48
BCR	15.8 (± 9.3)	11.2 (± 3.8)	21.4 (± 8.7)	0.023
Creatinine (mg/dl)	5.05 (± 5.2)	6.6 (3–9.7)	3.5 (0.71–4)	0.007
BUN (mg/dl)	51.3 (± 36.1)	62 (38–88)	40 (13–68)	0.037
Glucose (mg/dl)	119 (± 102)	99 (77–116)	137 (131–140)	0.02
Hemoglobin (g/dl)	12.09 (± 3.16)	11.6 (± 3.1)	12.5 (± 3.2)	0.78
WBC (cells × 10^3^)	7.4 (± 2.75)	8.0 (± 2.9)	6.8 (± 2.5)	0.37
Sodium (mmol/l)	135 (± 9.9)	132 (± 11.5)	137 (± 7.8)	0.34
Potassium (mmol/l)	4.4 (± 0.66)	4.4 (± 0.78)	4.5 (± 0.5)	0.6
Phosphorus (mg/dl)	5.4 (± 2.55)	6 (3–8.2)	4.6 (3.4–4.3)	0.14
Chlorine (mmol/l)	100 (± 10.6)	100 (± 13)	100 (± 7.5)	0.28
Magnesium (mg/dl)	2.1 (± 0.39)	2.1 (± 0.4)	2.1 (± 0.3)	0.36
*BCR* BUN to creatinine ratio, *BUN* Blood ureic nitrogen, *WBC* White blood cells				

Table 1 Demographic characteristics and biochemical variables at clinical diagnosis. Patient groups are divided according to the presence of AIN. Quantitative data are presented as the mean, maxima, and minima, whereas qualitative data contain a mean and percentage. The results were considered to be statistically significant when *p* < 0.05

The antecedent of recent drug consumption was present in 80% of patients with AIN and 60% in the group with no AIN. The most frequently used drugs were antihypertensives, such as losartan and telmisartan, as well as nonsteroidal anti-inflammatories (such as diclofenac and ketorolac) and analgesics, including tramadol. Concomitantly, two patients with AIN consumed herbal drugs for gastrointestinal disorders. Table 2 shows the complete details of the drugs that were ingested.

**Table 2:** Patient-reported drug therapy and herbalism

**Drugs**	**Herbalism**
**Antihypertensives** Losartan, telmisartan, furosemide, nifedipine, proponolol, valsartan, amlodipine, chlortalidone, and prazosine	*Eysenhardtia polystachya* *Ruta graveolesn*
**Antimicrobials/Antivirals** Ciprofloxacin, metronidazole, and tenofovir	
**NSAIDs/Analgesics** Paracetamol, diclofenac, ketorolac, Pregabaline, and tramadol	
**Antidiabetic** Insulin, metformin, and glibenclamide	
**Antiacids** Pantoprazole, omeprazole, and ranitidine	
**Others** Metoclopramide, tretinoine, allopurinol, and levotiroxine	

**Fig. 2:**
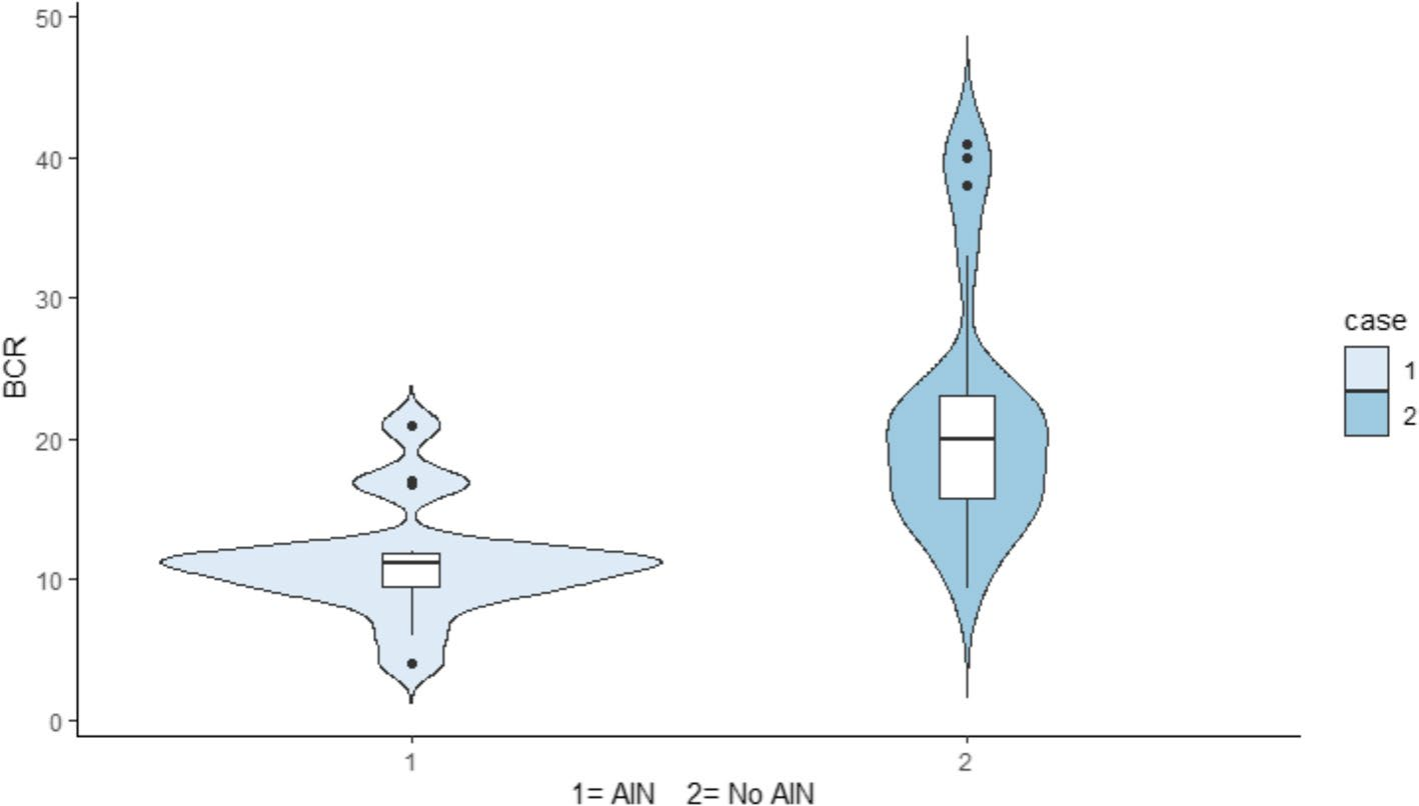
Violin plot showing the distribution of the BUN/creatinine ratio (BCR) in patients with acute kidney injury. In the AIN population, the median BCR is grouped at approximately 12

**Fig. 3:**
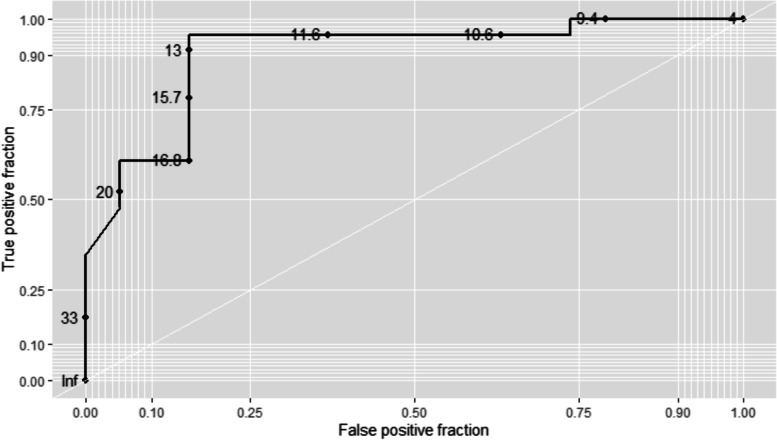
The ROC plot shows the optimal Youden point discriminating AIN from non-AIN patients with AKI as having a BCR value of 12. BCR = BUN to creatinine ratio

The bivariate analysis showed that patients with AIN had greater creatinine (6.6 mg/dL vs. 3.5 mg/dL) and BUN (62 mg/dL vs. 40 mg/dL) concentrations. The BCR was significantly lower in the AIN group (11.2 ± 3.8 vs. 21.4 ± 8.7, *p* = 0.001) (Fig. 2). The remaining clinical and biochemical variables were equally distributed between the groups, except for blood glucose concentrations, which were higher in the group with no AIN. The results of the multivariate analysis, BCR had an inverse correlation with the histological finding of AIN (Spearman rho = -0.57, *p* = 0.001). Table 3 shows the results of the multivariate analysis, with the variables suspected of being risk factors for AIN. Furthermore, the ROC analysis (Fig. 3) showed that the optimal Youden point for identifying AIN was at a BCR lower than 12 (sensitivity = 81%, specificity = 83%, and AUC = 0.73; *p* = 0.024).

**Table 3:** Results of logistic regression analysis

	**Estimate**	**Std Error**	**T value**	***p***
Intercept	1.202961	0.189453	6.350	0.001
BCR	0.025530	0.008493	3.006	0.004
BUN	-0.007325	0.004038	-1.814	0.07
Urea	0.004794	0.002619	1.831	0.07
Creatinine	-0.052293	0.025448	-2.055	0.06
Glucose	0.0002	0.004	1.2	0.4

The coexistence of AIN and acute tubular injury was observed in 12 patients, with a mean BCR of 12.1 ± 3. Nine patients had isolated acute tubular injury (without AIN), with a mean BCR of 17.5 ± 7. This difference was significant (*p* = 0.001). However, we did not identify an association between AIN and acute tubular injury (*p* = 0.2). In patients with glomerular disease, low BCR was observed only 13%; whereas in that with AIN, it was found in 84% (X^2^ = 21, *p* = 0.001).

BCR < 12 had good diagnostic performance for AIN (sensitivity = 76%, specificity = 81%, positive predictive value = 81%, and negative predictive value = 76%). Furthermore, BCR < 12 increased the probability of observing AIN in kidney histology (OR = 14.1, 95% CI = 2.6–75, *p* = 0.0021). In the multivariable analysis, BCR < 12 was the sole biomarker associated with histopathology-confirmed AIN. Individually, serum creatinine and BUN were not associated with AIN.

## Discussion

Our retrospective study demonstrated that low BCR (BCR < 12) adequately predicts the diagnosis of histological AIN in patients with AKI. The diagnostic utility of low BCR was previously described in obstructive uropathy, in which low BCR correlated with elevated urea in hospitalized patients [1, 16]. Additionally, BCR < 10 identified AIN in Mesoamerican nephropathy [4]. To our knowledge, there has been no other report on the diagnostic performance of BCR in a prospective study involving patients with AKI and AIN.

Creatinine is a product of muscle metabolism. Creatine is broken down into creatinine by a nonenzymatic mechanism. It is filtered in the glomerulus and secreted in the proximal tubule. At steady-state, the daily urinary creatinine that is excreted is equal to that produced, and this quantity is directly related to muscle mass [17]. In contrast, urea is excreted at the same rate as creatinine in the glomerulus but is reabsorbed into the tubules [18–20]. In the hemodynamic etiologies of AKI (prerenal), the activation by antidiuretic hormone causes preferential urea reabsorption, thus resulting in a BCR > 20 [20]. AIN is characterized by tubular dysfunction and an acute decrease in glomerular filtration rate. Edema, interstitial inflammation, and tubulitis are the main histopathological features of AIN [21]. In AIN, the abnormal reabsorption of water and sodium leads to increased urea excretion and decreased reabsorption [22]. The low BCR in patients with AIN can be explained by the combinatorial effects of the changes in urea excretion, filtration, and reabsorption with the reduction in creatinine secretion as the glomerular filtration rate declines.

The use of biomarkers in the diagnosis of AIN has been controversial in the literature. Novel biomarkers have focused on subclinical phases or prognostic factors in AKI. Neutrophil gelatinase-associated lipocalin (NGAL) is one of the most studied biomarkers. NGAL expression is related to stress or damage to the loop of Henle and the collecting duct; values greater than 104 ng/ml correlate with kidney damage [23, 24]. Another well-studied, novel biomarker is kidney injury molecule 1 (KIM-1), which is a transmembrane glycoprotein that is upregulated in response to ischemia‒reperfusion kidney injury [25]. Calprotectin is a heterodimer that is synthesized in epithelial cells in the collecting duct in response to damage or inflammation. Calprotectin is proposed to be able to distinguish intrinsic renal injury from prerenal etiology; however, its secretion is known to be increased in pyuria and some systemic inflammatory diseases (such as rheumatoid arthritis and inflammatory bowel disease) [24, 26] thus limiting the specificity of this novel biomarker. As a novel biomarker of AIN, BCR has multiple advantages, including low cost, ease of measurement, and the ability for longitudinal tracking of AIN. Creatinine and BUN are routinely measured in basic biochemical panels, and the establishment of a relationship between BCR and AIN would facilitate the diagnosis and early treatment of these patients prior to the analysis of renal histology.

A kidney biopsy is not systematically performed in patients with AIN. Drug-induced AIN can be diagnosed in patients who have a recent history of drug initiation, followed by subsequent creatinine elevation, urinalysis with white cells, white cell casts, eosinophiluria (in some cases), and symptom improvement after the cessation of the offending drug [27, 28]. However, histological confirmation is necessary for patients receiving drugs that are not known to precipitate AIN, with these patients lacking improvements in response to glucocorticoid treatment and having the absence of white cells in urinalysis. In addition, low BCR could help in the differential diagnosis of patients who are not improving with glucocorticoid treatment before the performance of a kidney biopsy. In this study, the BCR was calculated upon hospital admission of the AKI patient. The BCR can also vary over the course of the disease. Of note, BUN and creatinine can increase due to other etiologies, and it may be difficult to differentiate etiologies in patients with multiple factors contributing to AKI.

We acknowledge that this study was limited by a modest sample size and the study being performed at a single academic center. The study was also limited to hospitalized patients who consented to their participation, and the implications for outpatient AKI require further validation. Future experiments could involve further validation of the utility of the BCR in larger patient cohorts. These limitations could be overcome in future research by involving more recruiting centers and by increasing the etiologies of AIN. The first etiology of AIN was drug-induced; however, this information was obtained from anecdotal accounts from patients. This implied bias can be overcome by using databases with drugs prescribed to patients.

Our prospective study is the first of its kind to indicate a statistically significant correlation between BCR and histopathologically confirmed diagnosis of AIN. In addition, this prospective study was specifically designed to identify BCR as a predictor of AIN, which decreases the risk of bias.

## Conclusion

In hospitalized patients with AKI, the presence of BCR ≤ 12 is a robust parameter that suggests the diagnosis of AIN. The BCR is obtained from a basic metabolic panel, and its low cost allows for longitudinal quantification. Additionally, if urinalysis and clinical course are atypical for AIN, a low BCR could provide additional support and allow for the avoidance of kidney biopsy in some patients.

## Data Availability

The datasets used and/or analyzed during the current study are available from the corresponding author upon reasonable request.
